# Epigenetic marks and their relationship with BDNF in the brain of suicide victims

**DOI:** 10.1371/journal.pone.0239335

**Published:** 2020-09-24

**Authors:** Paulina Misztak, Patrycja Pańczyszyn-Trzewik, Gabriel Nowak, Magdalena Sowa-Kućma

**Affiliations:** 1 Department of Neurobiology, Maj Institute of Pharmacology, Polish Academy of Sciences, Kraków, Poland; 2 Chair of Pharmacobiology, Jagiellonian University Medical College, Krakow, Poland; 3 Department of Human Physiology, Institute of Medical Sciences, Medical College of Rzeszow University, Rzeszow, Poland; Chiba Daigaku, JAPAN

## Abstract

**Background:**

Suicide is a common phenomenon affecting people of all ages. There is a strong relationship between suicidal ideation and depressive disorders. Increasing number of studies suggest that epigenetic modifications in certain brain areas are the main mechanism through which environmental and genetic factors interact with each other contributing to the development of mental disorders. To verify this hypothesis, some epigenetic marks: H3K9/14ac, HDAC2/3, H3K27me2 and Sin3a, as well as p-S421-MeCP2/MeCP2 were examined. On the other hand, BDNF protein level were studied.

**Materials and methods:**

Western blot analysis were performed in the frontal cortex (FCx) and hippocampus (HP) of suicide victims (n = 14) and non-suicidal controls (n = 8). The differences between groups and correlations between selected proteins were evaluated using Mann-Whitney U-test and Spearman’s rank correlation.

**Results:**

Statistically significant decrease in H3K9/14ac (FCx:↓~23%;HP:↓~33%) combined with increase in HDAC3 (FCx:↑~103%;HP:↑~85% in HP) protein levels in suicides compared to the controls was shown. These alterations were accompanied by an increase in H3K27me2 (FCx:↑45%;HP:↑~59%) and Sin3a (HP:↑50%) levels and decrease in p-S421-MeCP2/MeCP2 protein ratio (HP:↓~55%;FCx:↓~27%). Moreover, reduced BDNF protein level (FCx:↓~43%;HP:↓~28%) in suicides was observed. On the other hand, some significant correlations (e.g. between H3K9/14ac and HDAC2 or between BDNF and p-S421-MeCP2/MeCP2) were demonstrated.

**Conclusions:**

Our findings confirm the role of epigenetic component and BDNF protein in suicidal behavior. Lowered BDNF protein level in suicides is probably due to decrease in histone acetylation and increased level of factors related with deacetylation and methylation processes, including MeCP2 factor, which may operate bidirectionally (an activator or inhibitor of transcription).

## Introduction

Suicide is an important public health issue of the 21^st^ Century. Each year, it affects around 800 thousand people globally [1]. According to the World Health Organization (WHO), suicidality is a principal reason of injury and mortality all over the world, ranking as the 2^nd^ leading cause of death in 15–29 year old group [2]. Suicidal behavior (SB) is conditioned by multiple factors which comprises complex relationships between character traits, psychosocial and environmental factors as well as genetic background [3, 4].

Among many risk factors for suicidality, mental disorders (especially affective disorders) seems to be the most critical. It is believed that up to 60% of those who commit suicide meet the diagnostic criteria for major depressive disorders (MDD). On the other hand, depression and suicide are also strongly interrelated [2]. Similarly, suicidal thoughts are significant cause of death in bipolar disorders (BD). Approximately 25–50% of BD patients undertake suicide attempts and 15% of these ends as successful [5]. Other psychiatric conditions (e.g., anxiety, psychotic and panic disorders, schizophrenia) are not insignificant in the development of SB as well [6]. Regardless of mental illness-based mechanisms, it seems that there are another independent brain factors contributing to suicides [4].

In the last dozen or so years, research on pathomechanisms of suicide and SB has been intensified. At the cellular level, numerous changes in the tissues of suicide victims have been demonstrated (see [7] for review). For example, our previous studies have revealed significant alterations in the N-Methyl-D-aspartate receptor (NMDAR) complex activity. A reduced affinity of zinc and magnesium to NMDAR accompanied by an increase in GluN2A and decrease of GluN2B and PSD-95 protein level in the hippocampus were shown [8]. Furthermore, in suicides was observed altered level of several proteins (zinc transporters; ZnTs) involving in the regulation of zinc homeostasis which was correlated with a decreased GluA1 and GluN2A expression in the frontal cortex [9]. Except that, numerous studies on the relationship between genetic factors and suicides point to changes in single genes [10]. What’s more, recent large genome-wide association studies (GWAS) have revealed certain genes (genetic variants) that predispose suicidal attempts or complete suicides [11–13]. On the other hand, numerous morphological changes (e.g. thinner prefrontal and anterior cingulate cortex) in the brains of suicides, as a consequence of altered expression and synthesis of neurotrophic factors, were described [14].

One of the most important neurotrophins, which plays critical role in the proper brain development and their functioning is the brain-derived factor (BDNF). It is hypothesized that pathological changes in *BDNF* expression are responsible for neuropsychological deficits related to stress, depression or suicide [15]. A reduced BDNF level both in the body fluids as well as brain structures was shown in the rodents subjected to various stress procedures [15–19]. Similarly, clinical studies was demonstrated lower serum or plasma BDNF concentration in patients diagnosed with major depression and manifesting suicidal behavior [15]. *Post mortem* studies indicate decreased BDNF level in the hippocampus and prefrontal cortex and no changes in the enthorinal cortex and the amygdala of completed suicides [19, 20]. On the other hand, in depressed patients downregulation of BDNF in anterior cingulate cortex, amygdala and caudal brainstem was reported [21, 22]. It should be emphasized that lower BDNF levels in anterior cingulate cortex were found in subjects who had been exposed to early life adversity and/or died by suicide in relation to nonsuicide decedents and no reported childhood adversity [3]. While the links between disturbed BDNF level and suicidal behavior are relatively well documented, the transcriptional regulation of BDNF is not fully explained [23].

Human (like rodent) *BDNF* gene contain nine exons (I-IX). It has been proven that each exon is regulated by its own unique promoter, conferring temporal and spatial control of *BDNF* expression in an activity-dependent manner. As a result, initially more than 10 different transcripts can be formed, but finally all of them are translated into an identical BDNF protein [23]. Changes in *BDNF* gene and further gene expression steps, such as modifications of the BDNF precursor protein, are also important for the end effect. In the context of mood disorders, two single nucleotide polymorphisms (SNPs): the Val66Met (also designed as: rs6265, G196A; located in the pro-BDNF region) and *BE5*.*2* (located in a cis-regulatory region controlling the activity of a *BDNF* promoter) that reduce evoked release of BDNF (e.g. in the hippocampus) seems to be the most important [24–26]. An increasing number of studies suggests that the *BDNF* gene expression can be effectively regulated by epigenetic modifications (without changes in DNA sequence). On the other hand, the role of epigenetic changes in the pathomechanism of mood disorders is increasingly confirmed [27, 28]. In this connection, understanding the epigenetic regulation of the *BDNF* expression can give a new light on the diagnosis and treatment of these disorders in the future [23, 29].

So far, DNA methylation is the most frequently studied epigenetic modification of the human genome in the context of mental disorders, including SB [30–33]. Among others, an associations between stress-related genes like *SKA2* (kinetochore-associated protein 2), *NR3C1* (Nuclear Receptor Subfamily 3 Group C Member 1), *GABA*_*A*_*α1 (*Gamma-aminobutyric acid A receptor *α1* subunit), *hGR* (human glucocorticoid receptor), *SAT1* (diamine acetyltransferase 1) or *HTR2A* (5-Hydroxytryptamine Receptor 2A) in brain multiple cohorts and suicidal behavior were demonstrated [34–38]. Further studies also showed a significant effect of hypermethylation of *BDNF* and tropomycin receptor kinase B (*TRKB*) promoters on their expression, resulting in brain plasticity dysfunction [39, 40]. Moreover, the increasing global methylation in the prefrontal cortex of suicide completers was also observed [4]. Interestingly, methylomic profiling of brain samples revealed differentially methylated regions (DMRs) in certain genetic loci (especially upstream of the *PSORS1C3* gene) associated with suicidal depression. More detailed study of these samples indicated hypomethylated areas within these DMRs and discrete modules of co-methylated loci related with polygenic risk burden only for suicide attempt (not major depression) [32]. On this basis, Murphy et al. (2020) conclude the existence of coordinated alterations in DNA methylation that are associated with SB [33].

On the other hand, numerous modifications of histones and enzymes involved in their formation have been described in *post mortem* brain tissues [32]. Distinct histone modifications are related with genomic features. For example, two histone marks: H3K4me3 (trimethylated lysine 4 of histone H3) and H3K9ac (acetylated lysine 9 of histone H3) are associated with active gene promoter, while repressed genes are characterized by H3K9 and H3K27 dimethylation or trimethylation [41].

Based on the available data, it can be concluded that epigenetic modifications are an important factors that critically influence gene expression (also those crucial for the proper functioning of the brain, like *BDNF*) and may have a significant impact on the appearance of suicide-related phenotypes. However, this problem requires a more detailed examination. So far, most studies on histone modification have concerned only predisposing genes while genome-wide approaches should be applied. Comprehensive study of histone modifications across the genome will allow to examine epigenetically altered neural circuits as the primary cause of suicide. In addition, examining the role of epigenetic mediators (such as MeCP2) will allow a better understanding of the nature of this phenomenon and interrelationships between individual epigenetic factors. Knowledge of the most important risk factors increasing the likelihood of committing suicide and its pathophysiology is indispensable for the formation of appropriate preventive systems.

The present study was undertaken to examine whether the several modifications of histones, and the enzymes involved in their formation (H3K9/14ac, H3K27me2, HDAC2, HDAC3, Sin3a) in the hippocampus and frontal cortex may play a role in suicidal behavior. Moreover, the MeCP2 protein level, which regulates genes (including BDNF) transcription by binding to methylated DNA and recruiting enzymes that modify histones, was studied. Finally, the level of BDNF protein was determined in the same brain tissues.

## Materials and methods

### Tissue collection

Brain tissues [Hippocampus (HP) and Frontal Cortex (FCx)–Brodmann area 10] were obtained from 14 non-diagnosed psychiatrically suicide victims (mean age ± SEM; 29.21 ± 3.594) and 8 unexpected sudden death controls (31.0 ± 4.89) at the time of autopsy in the Department of Forensic Medicine, Jagiellonian University Medical College [Grant no. 6P05B 142 20 from the State Committee for Scientific Research, approved by the Ethics Committee (2001–2004)]. The ethics committee approved a waiver of informed consent for this study knowing that participants will not and cannot be identified (we only have the data disclosed in this manuscript). The study subjects comprised 8 females and 14 males. According to the available medical history, both suicide and control subjects included in this study were not treated for any chronic central nervous system diseases (three cases of suicide death resulted from a single drug overdose). Demographic characteristics of each subject are described in Table 1.

**Table 1:** Demographic characteristics of controls and suicide subjects.

Group	Sex (male/female)	Age (years)	Cause of death
**Control**			
1	M	17	Cranial/brain injure
2	F	44	Road accident
3	M	20	Carbon monoxide poisoning
4	M	54	Myocardial infarction
5	M	29	Homicide
6	M	42	Myocardial infarction
7	F	21	Homicide
8	F	21	Road accident
**Suicide**			
1	M	33	Hanging
2	M	29	Hanging
3	F	21	Hanging
4	M	17	Hanging
5	M	47	Hanging
6	M	19	Jump under train
7	F	21	Self-poisoning/drug overdose (doxepine + clomipramine)
8	F	29	Jumping
9	M	20	Self-poisoning/drug overdose (hydroxyzine + perazine)
10	M	55	Hanging
11	F	20	Self-drowning
12	F	55	Self-poisoning/drug overdose (diazepam + ethanol)
13	M	24	Jumping
14	M	19	Hanging

M-male; F-female

### Western blot analysis

**Fig 1:**
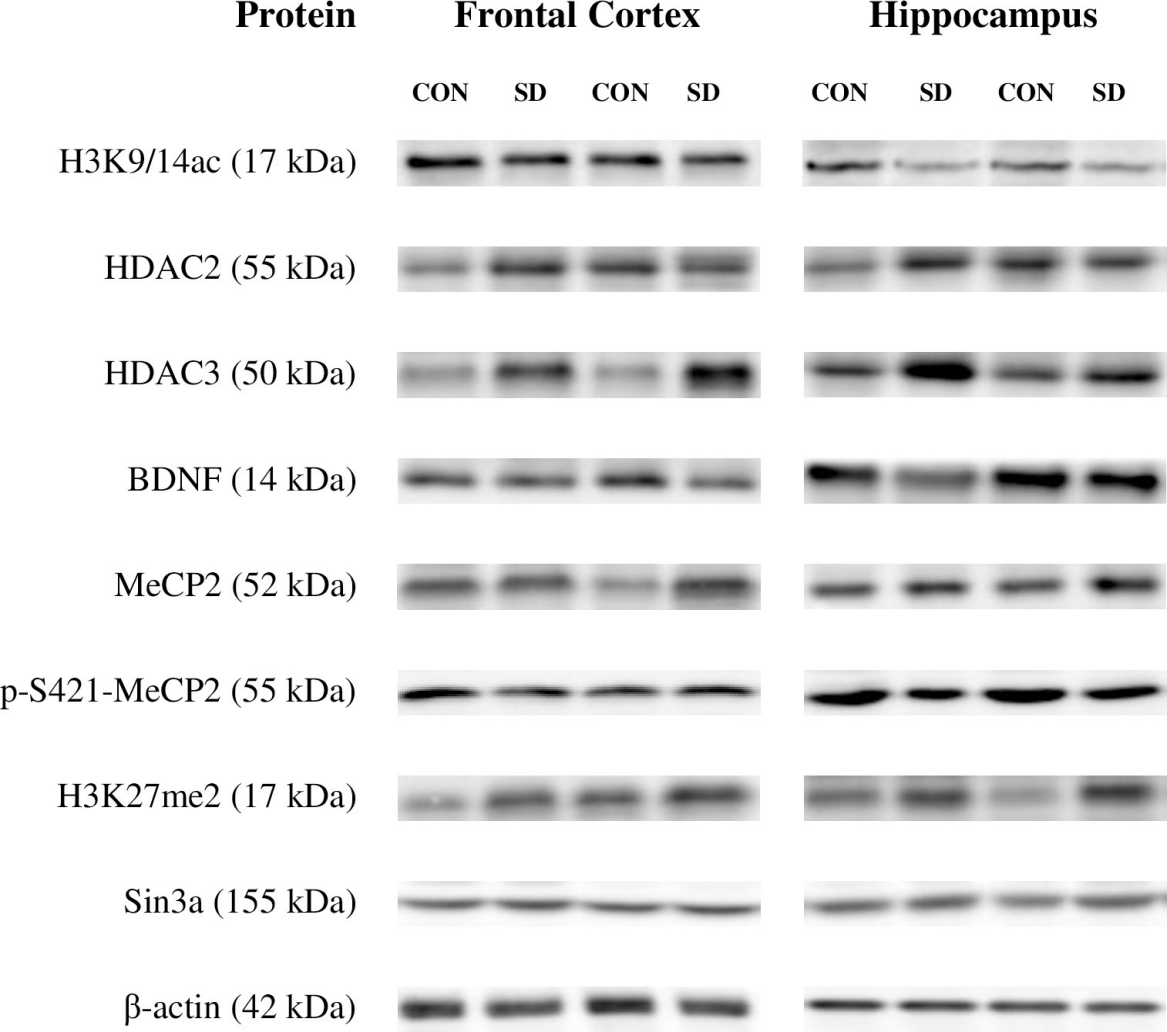
Immunoblots of MeCP2, p-S421-MeCP2, H3K27me2, Sin3a, H3K9/14ac, HDAC2, HDAC3, BDNF and β-actin from representative subjects used in the analysis. Each well was loaded with 30μg of total protein. *Abbreviations*: *CON–non-suicidal control; SD–suicide*.

The tissue samples were prepared according to the standard protocol [8]. After total protein determination by BCA method (Pierce Biotechnology, USA), samples containing 30 μg of total proteins and loading buffer were fractionated on 8–12% (depending on the molecular mass of analyzed protein) polyacrylamide gels and transferred to a nitrocellulose membrane (Bio-Rad, Germany). Non-specific signals were blocked using 1% blocking solution (BM Chemiluminescence Western Blotting Kit; Mouse/Rabbit, Roche, Switzerland). Next, the membranes were incubated overnight at 4°C with primary antibodies: anti-acetyl-Histone H3 (Lys9/14; H3K9/14ac) rabbit IgG antibody (Active Motif, USA; dilution 1:1,000); anti-HDAC2 mouse IgG2a (Cell Signaling, USA; dilution 1:1,000); anti-HDAC3 mouse IgG2a (Cell Signaling, USA; dilution 1:1,000); anti-BDNF rabbit antibody (Santa Cruz Biotechnology, USA; dilution 1:200); anti-MeCP2 rabbit polyclonal IgG (Santa Cruz Biotechnology, USA; dilution 1:300); anti-p-S421-MeCP2 rabbit polyclonal antibody (Abgent, USA; dilution 1:1,000); anti-di-methyl-Histone H3 (Lys27; H3K27me2) rabbit polyclonal antibody (Cell Signaling, USA; dilution 1:1,000) or anti-Sin3a rabbit antibody (Active Motif, USA; dilution 1: 1,000). After that, the membranes were washed 3 times for 10 min in Tris-buffered saline with Tween (TBS-T) and incubated at room temperature with goat anti-rabbit or anti-mouse IgG-HRP conjugated antibodies (Bio-Rad, Germany; dilution 1: 20,000) for 60 min. All antibodies used, both primary and secondary, were dissolved in 0.5% blocking solution (Roche, Switzerland). After incubation with secondary antibodies the blots were washed 4 times for 5 min with TBS-T and developed by enhanced chemiluminescence reaction (Roche, Switzerland). The protein signals were visualized and measured using a Fuji-Las 1000 system and Fuji Image Gauge ver. 4.0 software. As a control for transfer and loading, β-actin was assessed on each membrane. For this, mouse monoclonal anti-β-actin antibody (Sigma Aldrich, Germany; dilution 1:10,000) was used. Final results represent the ratio of the optical density of a particular protein to the optical density of β-actin present in the same sample. Representative Western blots are shown in Fig 1.

### Statistical analysis

The data was evaluated by GraphPad PRISM software (ver. 5.0, San Diego, CA, USA). The Shapiro-Wilk test was performed in order to evaluate the normal distribution of quantitative data. Because not all data showed the normal distribution we used the Mann-Whitney U-test to determine differences between studied groups. For the same reason, correlations between quantitative variables were assessed by Spearman’s rank correlation coefficient. To minimize the likelihood of a type 1 error, multiple testing correction was performed following Benjamini-Hochberg method. p<0.05 was considered as statistically significant.

## Results

Immunoreactive bands corresponding to molecular masses of 17, 55/50, 14, 55/52, 17, 155, and 42 kDa were revealed for H3K9/14ac, HDAC2/3, BDNF, p-S421-MeCP2/MeCP2, H3kK27me2, Sin3a and β-actin, respectively (Fig 1).

**Fig 2:**
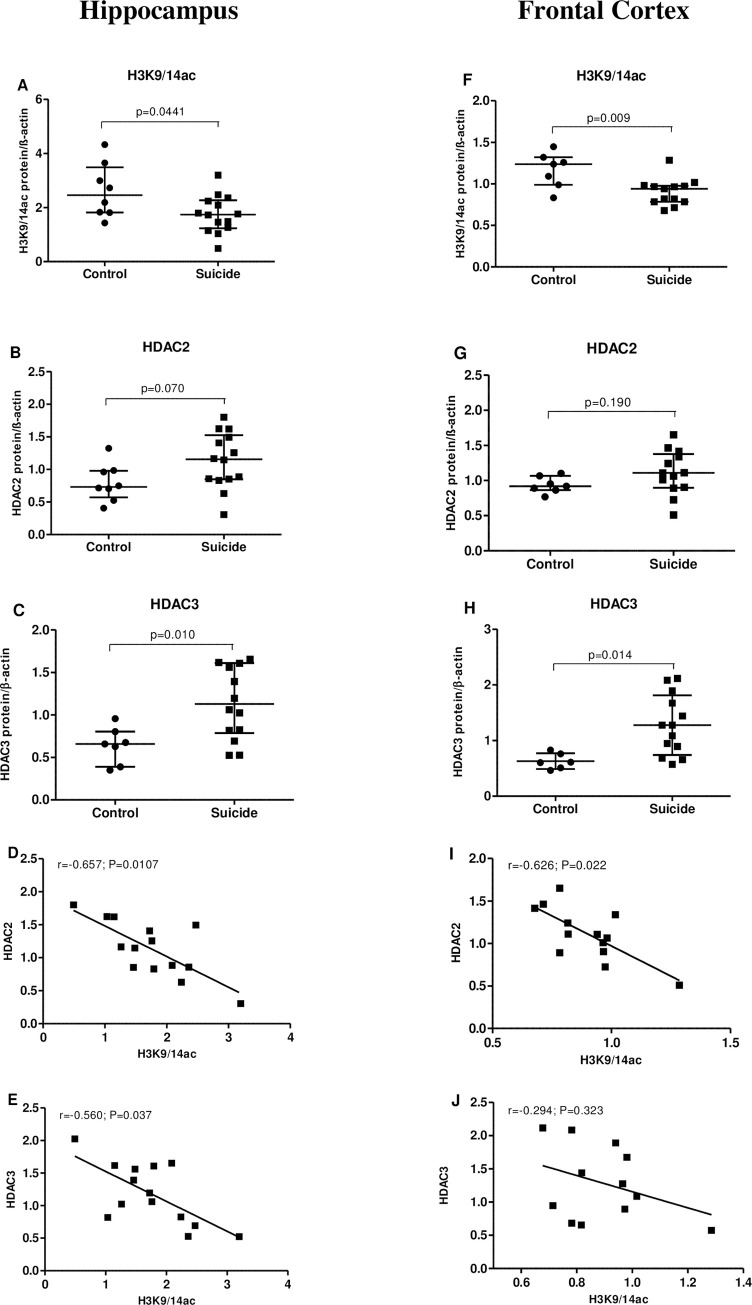
Correlation between H3K9/14ac and HDAC2/3 in the hippocampus (left panel; A-E) and frontal cortex (right panel; F-J) of suicide victims. Relative protein level of H3K9/14ac (A, F), HDAC2 (B, G) and HDAC3 (C, H) in non-suicide control subjects (n = 8) and suicide victims (n = 14). Data are expressed as investigated protein levels in relation to β-actin (mean R.O.D values ± SEM). Correlation analysis for the H3K9/14ac and HDAC2 (D, I) and H3K9/14ac and HDAC3 (E, J) in suicide victims.

As shown in Fig 2, the amount of H3K9/14ac immunoreactivity from suicide victims was significantly lower than that of the control subjects, both in HP [↓33.1%, p = 0.0441, Mann-Whitney U-test] (Fig 2A) and FCx [↓22.8%, p = 0.009, Mann-Whitney U-test] (Fig 2F). Conversely, there was a robust increase in the HDAC3 protein level [HP: ↑85.3%, p = 0.010 (Fig 2C); FCx: ↑102.8%, p = 0.014 (Fig 2H), Mann-Whitney U-test] and a slight, but insignificant increase in HDAC2 protein level (Fig 2B and 2G, respectively) in suicides. Interestingly, our results showed significant correlations between H3K9/14ac and HDAC2 protein level in the both studied brain regions of suicide victims [HP: r = -0.657, p = 0.0107; FCx: r = -0.626, p = 0.022; Spearman’s rank correlation] (Fig 2D and 2I, respectively). Furthermore, there was significant correlation between H3K9/14ac and HDAC 3 protein level in the hippocampus [r = -0.560, p = 0.037; Spearman’s rank correlation] (Fig 2F), but not in the frontal cortex (Fig 2J).

**Fig 3:**
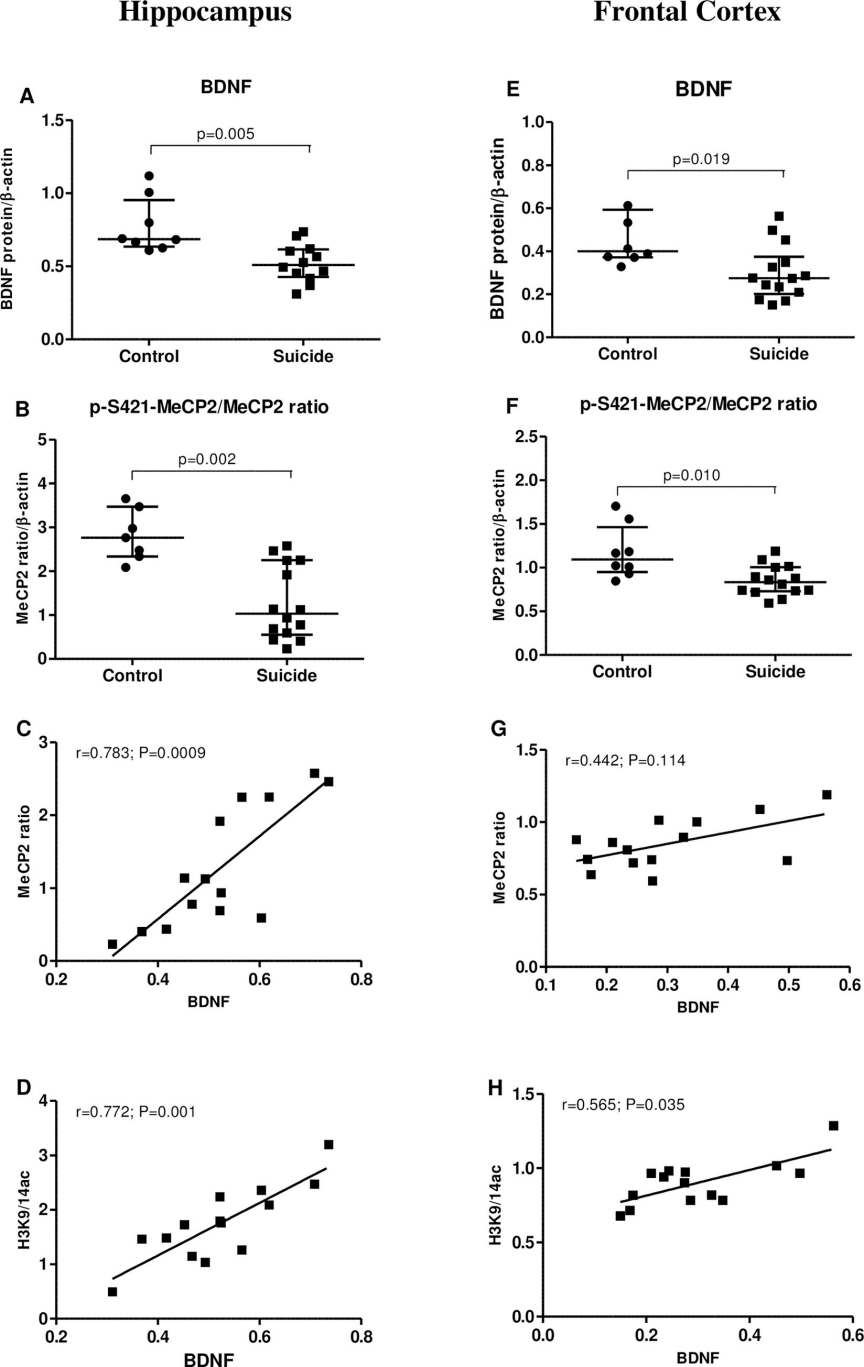
Correlation between BDNF and H3K9/14ac or p-S421-MeCP2/MeCP2 ratio in the hippocampus (left panel; A-D) and frontal cortex (right panel; E-H) of suicide victims. Relative protein level of BDNF (A, E) and p-S421-MeCP2/MeCP2 (B, F) in non-suicide control subjects (n = 8) and suicide victims (n = 14). Data are expressed as investigated protein levels in relation to β-actin (mean R.O.D values ± SEM). Correlation analysis for the BDNF and p-S421-MeCP2/MeCP2 (C, G) and BDNF and H3K9/14ac (D, H) in suicide victims.

Subsequent analysis revealed significant decrease in BDNF protein level of suicide group compared to the matched controls in HP [↓28%, p = 0.005, Mann-Whitney U-test] (Fig 3A) and FCx [↓42.03%, p = 0.019, Mann-Whitney U-test] (Fig 3E). Simultaneously, significant reduction in p-S421-MeCP2/MeCP2 protein ratio were demonstrated [HP: ~55%, p = 0.002 (Fig 3B); FCx: ~27%, p = 0.010 (Fig 3F), Mann-Whitney U-test]. Our findings also indicated significant positive correlations between BDNF level and p-S421-MeCP2/MeCP2 protein ratio in the hippocampus of suicide victims [r = 0.783, p = 0.0009 (Fig 3C)] and also between BDNF and H3K9/14ac protein levels, both in HP [r = 0.772, p = 0.001 (Fig 3D); Spearman’s rank correlation] and FCx [r = 565, p = 0.035 (Fig 3H), Spearman’s rank correlation].

**Fig 4:**
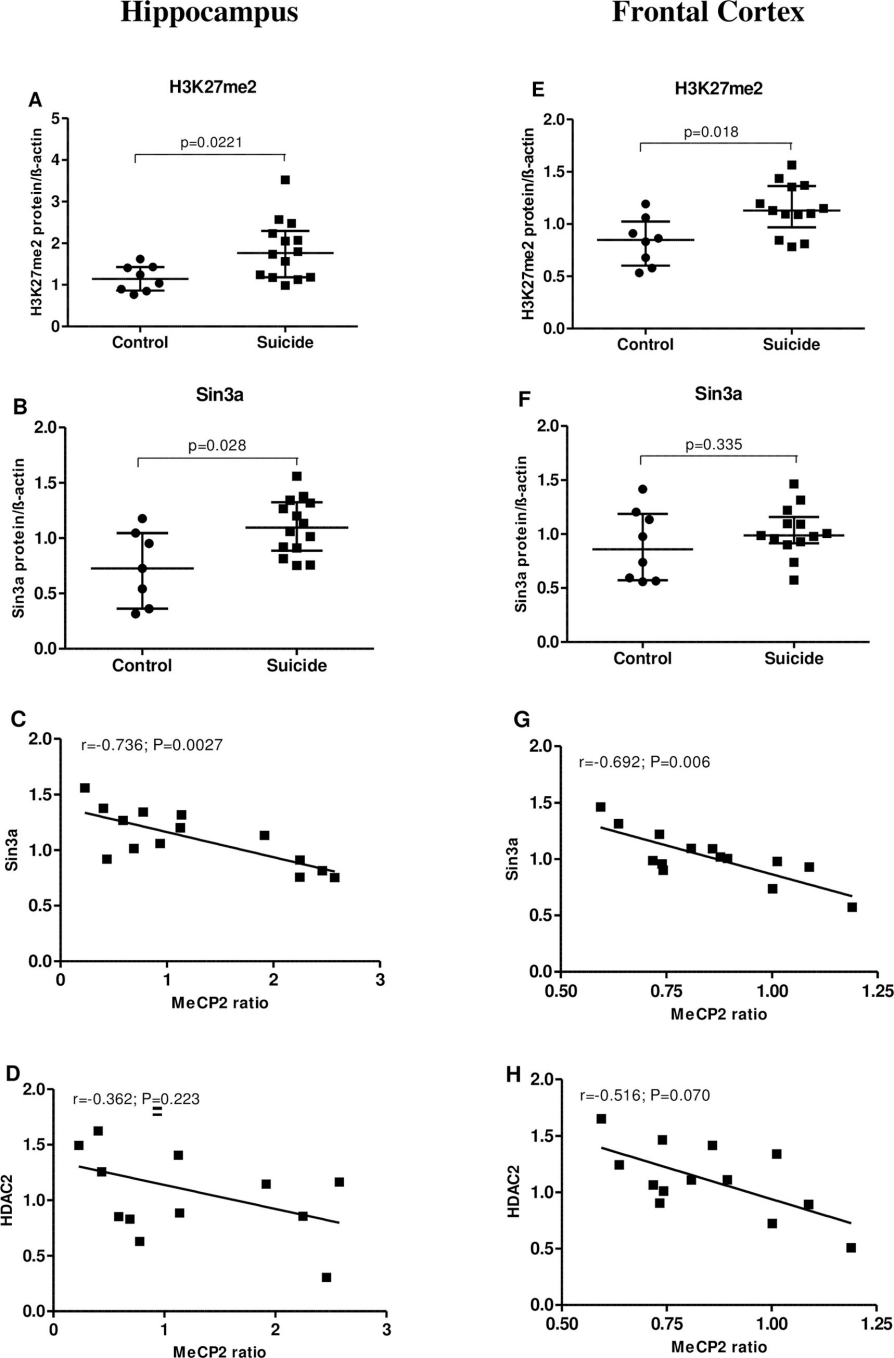
Correlation between p-S421-MeCP2/MeCP2 ratio and Sin3a or HDAC2in the hippocampus (left panel; A-D) and frontal cortex (right panel; E-H) of suicide victims. Relative protein level of H3K27me2 (A, E) and Sin3a (B, F) in non-suicide control subjects (n = 8) and suicide victims (n = 14). Data are expressed as investigated protein levels in relation to β-actin (mean R.O.D values ± SEM). Correlation analysis for the MeCP2 ratio and Sin3a (C, G) and MeCP2 ratio and HDAC2 (D, H) in suicide victims.

What’s more, significant changes in the H3K27me2 [HP: ↑58.9%, p = 0.0221 (Fig 4A); FCx: ↑45.0%, p = 0.018 (Fig 4E), Mann-Whitney U-test] and Sin3a [HP: ↑50.0%, p = 0.028 (Fig 4B), Mann-Whitney U-test] protein levels of suicide victims when compared to the sudden death controls were noted. Spearman’s rank correlation revealed strong negative correlation between MeCP2 ratio and Sin3a level, both in the hippocampus [r = -0.736, p = 0.0027 (Fig 4C); Spearman’s rank correlation] and frontal cortex [r = -0.692, p = 0.006 (Fig 4G); Spearman’s rank correlation], and no significant relationship between HDAC2 protein and MeCP2 ratio (Fig 4D and 4H).

## Discussion

A first major finding of this study is that suicidal behavior is accompanied by decreased histone acetylation combined with changes in HDAC2/3 protein level, both in the frontal cortex and hippocampus of *post mortem* brain.

Typically, histone acetylation is related to gene activation. Addition of acetyl group to N-terminal lysine, especially on histone H3 or H4, leads to chromatin relaxation and allows the transcriptional machinery to initiate gene expression. These enzymatic reaction is catalysed by acetyltransferases (HATs), while removal of acetyl group from histone tails, condensation of chromatin and silencing of gene transcription are accelerated by histone deacetylases (HDACs) [28]. Genome-wide studies have shown that histone acetylation mainly concern gene promoters [42]. In our work, we decided to examine alterations in global levels of H3K9/14ac protein. A large body of evidence has suggested that histone acetylation in several brain structures has an overall adaptive role in stress and antidepressant responses. Covington et al. showed that histone acetylation (H3K14ac) is first temporarily lowered and next persistently increased in the nucleus accumbens (NAc) after chronic social defeat stress, and that this is correlated with a reduction in HDAC2 levels [43]. Similar changes were also indicated in the NAc of depressed patients in *post mortem* investigation [42]. In the hippocampus, contrary to what was observed in the NAc after chronic social defeat stress, there was (following a transient increase) a sustained decrease in H3K14ac. These alterations were reversed by antidepressant (imipramine) treatment [44]. Similarly, in rats subjected to the chronic unpredictable or variable stress model lowered H3 or H4 acetylation on lysine 9 or 12 (respectively) were observed [45, 25–46]. On the other hand, exposition to some acute stressors resulted in an increase in histone (phospho)acetylation in certain subareas of HP in rodents [47, 48].

NAc and HP are most widely studied brain structures in the context of mood disorders and epigenetics together, whereas only a few papers concern changes in other regions of the cortical-striatal-limbic circuit in the brain. In the amygdala, increased H3K14ac level in the chronic social defeat stress model in mice (both 1 and 24 f after the last defeat) was demonstrated. After longer periods H3 acetylation returned to baseline [44]. Interestingly, acute social defeat stress in rats reduced transiently H3 acetylation in the amygdala, while there was no effect of chronic stress [49]. Simultaneously, global levels of histone acetylation in the prefrontal cortex do not seem to change after acute or chronic stress in rodent models [44, 49]. On the one hand, it seems to be in opposition to our results, but on the other hand, it may suggest that suicidal behavior is a consequence of another adverse factors, hence their effect on the brain structures may be different.

In the light of the above data, it can be suggested that histone hypoacetylation in the brain is a common characteristic of mood disturbances and its hyperacetylation (especially H3) may be an indicator of the antidepressant activity of compounds. However, it must not be forgotten that any changes in chromatin acetylation have serious consequences in the activity of key genes. In present study we showed that decreased level of H3K9/14ac, both in the HP and FCx was positively correlated with the BDNF protein. The role of BDNF in both pathophysiology and therapy of depressive disorders seems to be strongly confirmed [50, 51]. Similarly, the relationship between BDNF and suicidal behavior is well documented [3, 19, 20, 52]. Most published data indicate a significant decrease in BDNF levels in suicides, which is why our observations are consistent with previous studies [3, 19, 20, 53, 54]. Although BDNF seems to be a promising marker of suicidal behavior, it should be remembered that its expression in various tissues (including brain) may be region-specific [3, 19, 20]. Similarly, some differences between blood components were observed in suicides [52]. Altered BDNF level in suicides may be the consequence of persistent (like Val66Met polymorphism) or variable genome changes (like higher methylation status in *BDNF* gene promoter or changed histone code due to post-translational changes in chromatin) (see [55]).

The relationship between histone acetylation and *BDNF* expression has been the subject of numerous previous studies. For example, Zheng et al. revealed that decreased of H3K14 acetylation as well as increased of HDAC2 and HDAC1 levels are related to downregulation of hippocampal BDNF expression in offspring from gestational stress dams [56]. Contrary, after electroconvulsive shock (ECS) treatment, acetylation of H3 (but not H4) was increased which was necessary to regulate BDNF expression [57]. There is also proven the influence of histone H3 acetylation on NMDA/ERK/MSK1/2 pathway which is related with BDNF synthesis [58].

Our work revealed lowered H3K9/14ac accompanied by an increase in the HDAC2 and HDAC3, both in FCx and HP of suicide victims as compared to the sudden death controls. Previously, it was shown a decline in the HDAC2 protein in the nucleus accumbens (NAc) in clinical depression [59]. In turn, other authors found an elevated HDAC2 and HDAC5 mRNA level in white blood cells of MDD patients in the depressive episode [43]. Even more varied changes in HDACs expression pattern were revealed in the studies on animal models of depression and after antidepressant administration (see [28] for review). It is well known that HDAC2 plays essential role in the development, cell cycle progression and gene regulation whereas HDAC3 can downregulate p53 (nuclear transcription factor) and thereby modulate cell growth and apoptosis [60]. It seems that in suicides acetylation of H3 is downregulated but on the other hand HDAC 3 and 2 are upregulated. In the frontal cortex HDAC2 and HDAC3 may play role in genes silencing and apoptosis exaggeration. Meanwhile in the hippocampus HDAC3, but not HDAC2, may regulate gene which are responsible for adaptation of central nervous system, but it need to be examined. Furthermore, downregulation of acH3 and upregulation of HDAC2/3 may be corresponding with each other and be bound with methylation processes.

Another important findings of this study is that suicidal behavior is characterized by serious changes in histone H3 methylation (H3K27me2) status as well as chromatin-associated protein (MeCP2/p-S421-MeCP2) which binds to region of methylated CpG in genome and play essential role in the gene expression. MeCP2 (*Methyl-CpG-Binding Protein 2*) has two domains, one recognize methylated cytosine (5mC)–MBD and another terminate the transcription—TRD, so this molecule may be viewed as transcriptional modulator. MeCP2 is functionally associated with a variety of signal transduction pathways in the cell and plays a key role in the maturation of neurons, synaptogenesis and nervous system development. In response to neural stimulation, MeCP2 is phosphorylated on serine 421 (S421), leading to its activation [61]. It was found that MeCP2, depending on the current needs of neuron, acts as a repressor or activator of transcription [62]. Knowledge of epigenetic and metabolic mechanisms by which MeCP2 protein controls the expression of target genes is still restricted. There is evidence that in resting neurons MeCP2 regulates gene expression by binding to methylated CpG dinucleotides in gene promoters, where it forms complexes with other repressors, including histone deacetylases (e.g., HDAC1, HDAC2) and histone methyltransferases (HMT), and co-repressor Sin3a. As a consequence, chromatin condenses, limiting the access of transcriptional factors and enzymes to gene promoters. Conversely, neuronal activation evokes MeCP2 phosphorylation which results in its release from the promoter region and dissociation of the corepressor complex [63]. Due to the fact that MeCP2 can actively binds to methylated DNA as well as causes further modification of chromatin (alone or by recruiting other factors), this protein is called multi-talented epigenetic factor or global chromatin regulator [64]. Importantly, the existence of numerous phosphorylation (and other post-translational modifications) sites within the polypeptide chain of MeCP2 allow very precise regulation of its activity and adaptation to current needs of the cell [65]. Mutations in the gene encoding MeCP2 are associated with Rett syndrome, Angelman syndrome, mental retardation, MDD and addiction [66, 67]. In cocaine addiction, MeCP2 selectively regulate *BDNF*, *CBP* (CREB-binding protein) and *CAMK2d* (calcium/calmodulin dependent protein kinase II delta) genes transcription [68]. The mechanism of MeCP2-dependent regulation of the *BDNF* gene suggests enhanced BDNF expression in the absence of MeCP2 [65].

MeCP2 is probably multidimensionally involved in the pathophysiology of depression. Hutchinson et al. revealed crucial role of MeCP2 phosphorylation (p-MeCP2-Ser421) in antidepressant properties of citalopram and imipramine. Increasing level of MeCP2 caused depressive behavior and lack of S421 phosphorylation lead to absence to antidepressant action of this drugs [69, 70]. Moreover, p-MeCP2-Ser421 is necessary for deactivation of repressive HDAC/Sin3a complex and activation of transcription [63]. Neuronal activation is necessary for MeCP2 phosphorylation which lead to hyperacetylation and releasing of chromatin [62]. These fact suggest that MeCP2 and methylation of DNA may induce changes in acetylation/deacetylation pattern. MeCP2 also interacts with Sin3a and H3K27me2. Sin3a is a transcription regulator which works together with REST (Restrictive Element-1 Silencing Transcription Factor) corepressor and regulates cell cycle progression and gene inhibition. Di-methylation (me2) at histone H3 lysine 9/27 (H3K9/K27) is related with euchromatic gene repression. Genome-wide studies have revealed a widespread H3K9/K27 methylation alterations at the promoters of many genes in two animal models of depression (social defeat and social isolation), the majority of which are reversed by chronic antidepressant treatment [42, 71]. Another study showed that an increase in the H3K27me2 level was correlated with decreased BDNF expression in the hippocampus after chronic social defeat stress, and this induction of repressive methylation was persistent despite chronic imipramine treatment [72]. The association of H3 methylation with reduced BDNF level in the brain was also confirmed by other authors [42, 73].

In our work, an increase of H3K27me2, and decrease of p-MeCP2-Ser421/MeCP2 protein ratio in suicide tissue was observed. Interestingly, in the hippocampus (but not in the frontal cortex) elevated Sin3a protein level was also shown. On the other hand, a significant negative correlation between the level of MeCP2 and Sin3a was revealed in both examined structures, which indicates the complexity of epigenetic interactions in the brain. It seems that in the hippocampus and frontal cortex epigenetic machinery operates in a different structure-dependent manner. Although we have noted a decreased BDNF level both in FCx and HP, it seems that BDNF expression is regulated by different epigenetic factors depend to structure.

According to our knowledge in HP BDNF may be regulated by corepressor complex with MeCP2/HDAC3/Sin3a and decreasing level of p-MeCP2-Ser421 may suggest role of BDNF and neuronal silencing by decreased neuronal activity and gene transcription. In the frontal cortex BDNF is also reduced but it seems to be the effect of gene inhibition based on its methylation. Considering the above knowledge and data from studies indicating both an increase in global DNA methylation [4] and the presence of hypomethylated regions at selected genetic loci in suicides [32, 33], it seems justified and necessary to further investigate the role of MeCP2 in the context of mood disorders. HDAC2 increased in HP impaired special memory and synaptic plasticity [74] and with HDAC5 plays important role in MDD patients [75]. We suggest that in FCx HDAC2 may regulate different genes not only BDNF but also involved on cortical abnormalities and metabolic impaired but it need to be examined.

In summary, our study, for the first time describe alterations in the BDNF levels in suicide victims on the background of serious and multidimensional epigenetic changes both in the frontal cortex and hippocampus. The obtained results contribute to a better understanding of the mechanisms underlying the suicidal behavior. On the other hand, we are fully aware of some limitations of this study, especially those related to the subjects’ description. First of all, based on the available demographic characteristics, we do not know whether suicide victims suffered from depression. We only have information that suicides included in the study were not treated for depression, which is not synonymous with the fact that they did not suffer from it. On the other hand, it is estimated that about 60% of suicides met the diagnostic criteria for MDD [2]. Furthermore, it has been revealed that suicide completers more likely suffered from MDD than suicide attempters [9]. This allows us to conclude about the potential coexistence of depressive disorders in the study subjects for whom no diagnostic information was available. The support of this hypothesis may be the results of our previous study, which showed a similar direction of biochemical changes in the prefrontal cortex of patients diagnosed with MDD and suicides (with limited medical history) [9]. On the other hand, lacking of psychiatric diagnosis for the suicide victims, it cannot be ruled out that the observed epigenetic changes are a consequence of other factors than depression.

Equally important, three of the fourteen suicide subjects died from a drug overdose. We assume that the use of these drugs (in the following combinations: doxepine + clomipramine; hydroxyzine + perizine; diazepam + ethanol) was incidental and the suicide completers had not previously used these drugs regularly. However, it is worth noting that at least one of these three cases used a combination of typical antidepressant drugs. Although, comparison of the results obtained from these 3 subjects (alone or in combination) with the results obtained from the remaining 11 tissues did not indicate statistically significant differences (results not presented), we cannot exclude the possibility that changes in the levels of the studied proteins may result (at least partially) from the effects of drug medications.

Another limitation of this study is also the lack of information on potential comorbidities, postmortem interval (PMI) or pH of tissues, which could also critically affect the final result of this study.

Despite the above-mentioned most important limitations of this study, we believe that presented results will contribute to deepening our understanding of the role of epigenetic mechanisms in suicidal behavior and shed new light on previous research.

## Conclusions

Our findings confirm the role of epigenetic component and BDNF protein in suicidal behavior. Lowered BDNF protein level in suicides is probably due to decrease in histone acetylation and increased level of factors related with deacetylation and methylation processes, including MeCP2 factor, which may operate bidirectionally (an activator or inhibitor of transcription).

The mechanisms of epigenetic regulation of gene expression in the brain seems to be very complicated, multidirectional and structure-dependent, therefore, further, extended studies should be undertaken to find reliable epigenetic markers associated with suicide.

## Supporting information

S1 Raw images (PDF)

## Abbreviations

BDNF: *brain-derived neurotrophic factor*
H3K9/14ac: *histone H3 acetylated at lysines 9 and 14*
HDAC2/3: *histone deacetylase 2/3*
MeCP2: *Methyl-CpG-binding protein 2*
p-S421-MeCP2: *MeCP2 phosphorylated at serine 421*
H3K27me2: *dimethylated histone H3 at lysine 27*
Sin3a: *SIN3 transcription regulator family member A*
FCx: *frontal cortex*
HP: *hippocampus*
