# Supplementary material for: Epigenetic marks and their relationship with BDNF in the brain of suicide victims
Source: PLoS One. 2020 Sep 24;15(9):e0239335. doi: 10.1371/journal.pone.0239335 (PMC7513998; doi:10.1371/journal.pone.0239335)

Western blot analysis – methodology. Qualitative analysis of blots.

The tissue samples were prepared according to Sowa-Kucma et al. [2013]. After total protein determination by BCA method (Pierce Biotechnology, USA) using Synergy HTX multiplate reader (BioTek, USA). Samples containing 30 µg of total proteins and loading buffer (Novex Tris-Glycine SDS Sample Buffer 2x, Invitrogen) were fractionated on 8-12% (depending on the molecular mass of analyzed protein) polyacrylamide gels using Mini-Protean Tetra Vertical Electrophoresis Cell (Bio-Rad, Germany). Both controls and suicide samples were fractionated on one gel. Due to the limited number of wells in the gel, the samples were fractionated on different gels in different configurations. After electrophoresis, a wet transfer of proteins to a nitrocellulose membrane was made (Bio-Rad, Germany). Non-specific signals were blocked using 1% blocking solution (BM Chemiluminescence Western Blotting Kit; Mouse/Rabbit, Roche, Switzerland). Typically, after blocking, membranes were cut into smaller fragments [according to the protein size marker; two different protein ladder were use in this study: Spectra™ Multicolor Broad Range Protein Ladder (ThermoFisher Scientific) or BenchMark Protein Ladder (Invitrogen)] maintaining a sufficiently large fragment beyond the analyzed band. The membranes were incubated overnight at 4°C with primary antibodies: anti-acetyl-Histone H3 (Lys9/14; H3K9/14ac) rabbit IgG antibody (Active Motif, USA; dilution 1:1,000); anti-HDAC2 mouse IgG2a (Cell Signaling, USA; dilution 1:1,000); anti-HDAC3 mouse IgG2a (Cell Signaling, USA; dilution 1:1,000); anti-BDNF rabbit antibody (Santa Cruz Biotechnology, USA; dilution 1:200); anti-MeCP2 rabbit polyclonal IgG (Santa Cruz Biotechnology, USA; dilution 1:300); anti-p-S421-MeCP2 rabbit polyclonal antibody (Abgent, USA; dilution 1:1,000); anti-di-methyl-Histone H3 (Lys27; H3K27me2) rabbit polyclonal antibody (Cell Signaling, USA; dilution 1:1,000) or anti-Sin3a rabbit antibody (Active Motif, USA; dilution 1: 1,000). As a control for transfer and loading, β-actin was assessed on membrane. For this, mouse monoclonal anti-β-actin antibody (Sigma Aldrich, Germany; dilution 1:10,000) was used. After incubation with primary antibodies, the membranes were washed 3 times for 10 min in Tris-buffered saline with Tween (TBS-T) and incubated at room temperature with goat anti-rabbit or anti-mouse IgG-HRP conjugated antibodies (Bio-Rad, Germany; dilution 1: 20,000) for 60 min. All antibodies used, both primary and secondary, were dissolved in 0.5% blocking solution (Roche, Switzerland). After incubation with secondary antibodies the blots were washed 4 times for 5 min with TBS-T and developed by enhanced chemiluminescence reaction (Roche, Switzerland). The protein signals were visualized and measured using a Fuji-Las 1000 system. Images were saved as

.img file format and next quantified using Fuji Image Gauge ver. 4.0 software. In order to prepare the documentation for publication, the images were exported to .tif format. Final results represent the ratio of the optical density of a particular protein to the optical density of β-actin present in the same sample. To limit the amount of biological material used in the study, some membranes were stripped and reused (re-incubation with antibodies) to identify other proteins on the same membrane. Such a solution was practiced only for proteins of different molecular weight (bands at different levels of the membrane).

References:

[1] Sowa-Kućma M, Szewczyk B, Sadlik K, Piekoszewski W, Trela F, Opoka W, Poleszak E, Pilc A, Nowak G. Zinc, magnesium and NMDA receptor alterations in the hippocampus of suicide victims. J Affect Disord 2013;151:924-31.

Legend for figures:

- Controls – samples: C1, C3, C4, C7, C11, C15, C16, C17
- Suicides – samples: S2, S5, S6, S8, S9, S10, S12, S13, S14, S18, S19, S20, S21, S22
- X – sample excluded from analysis

HIPPOCAMPUS

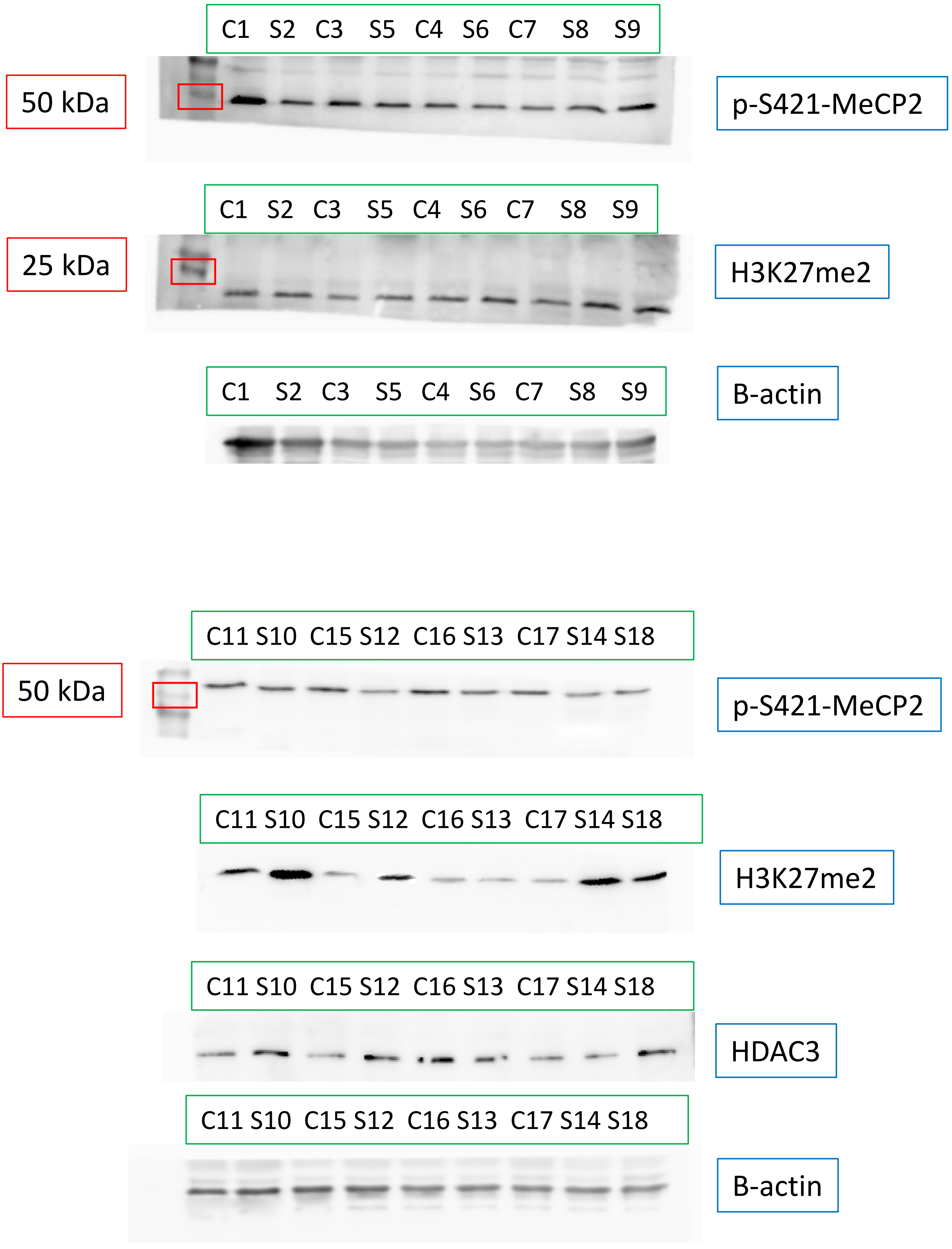

HIPPOCAMPUS

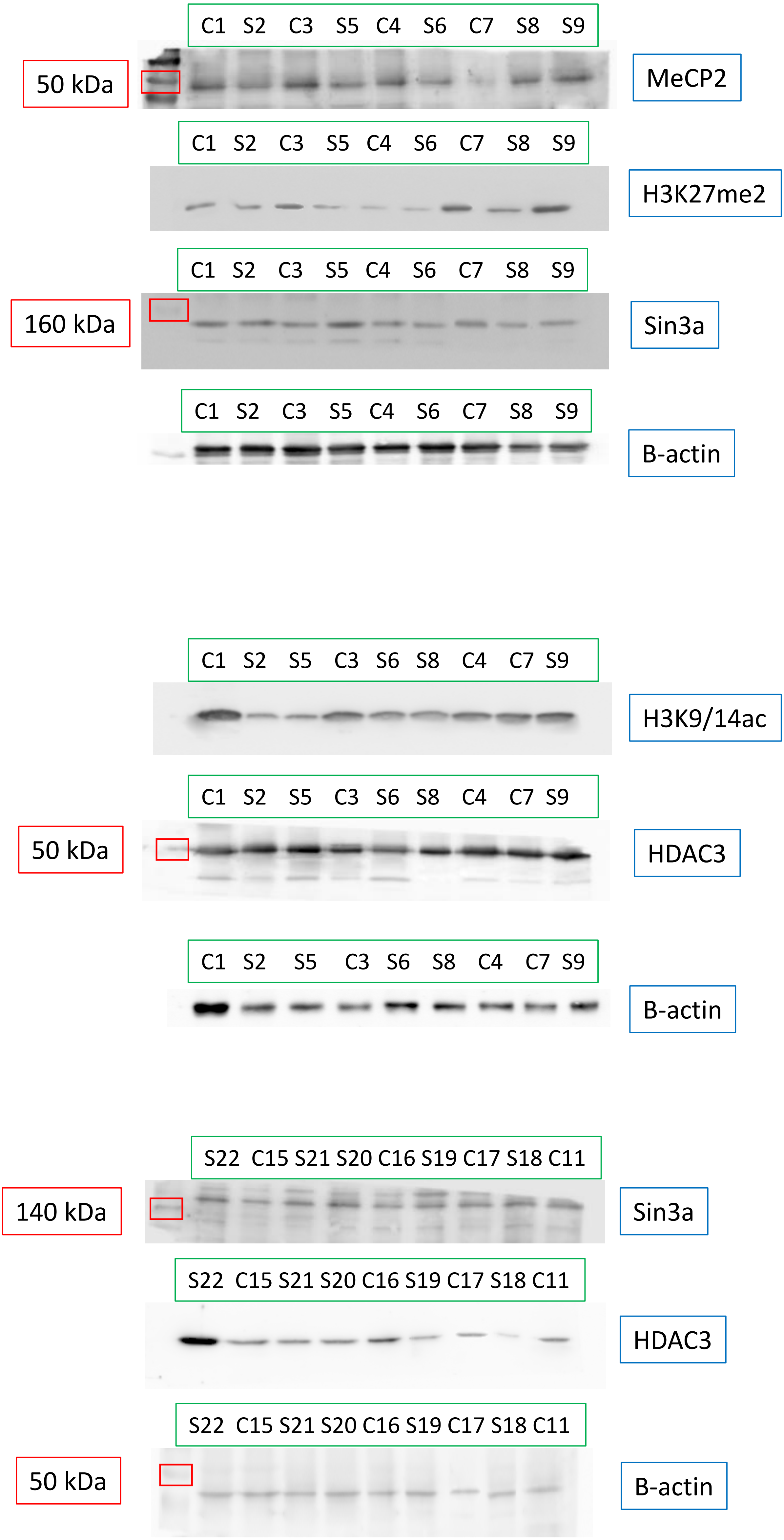

HIPPOCAMPUS

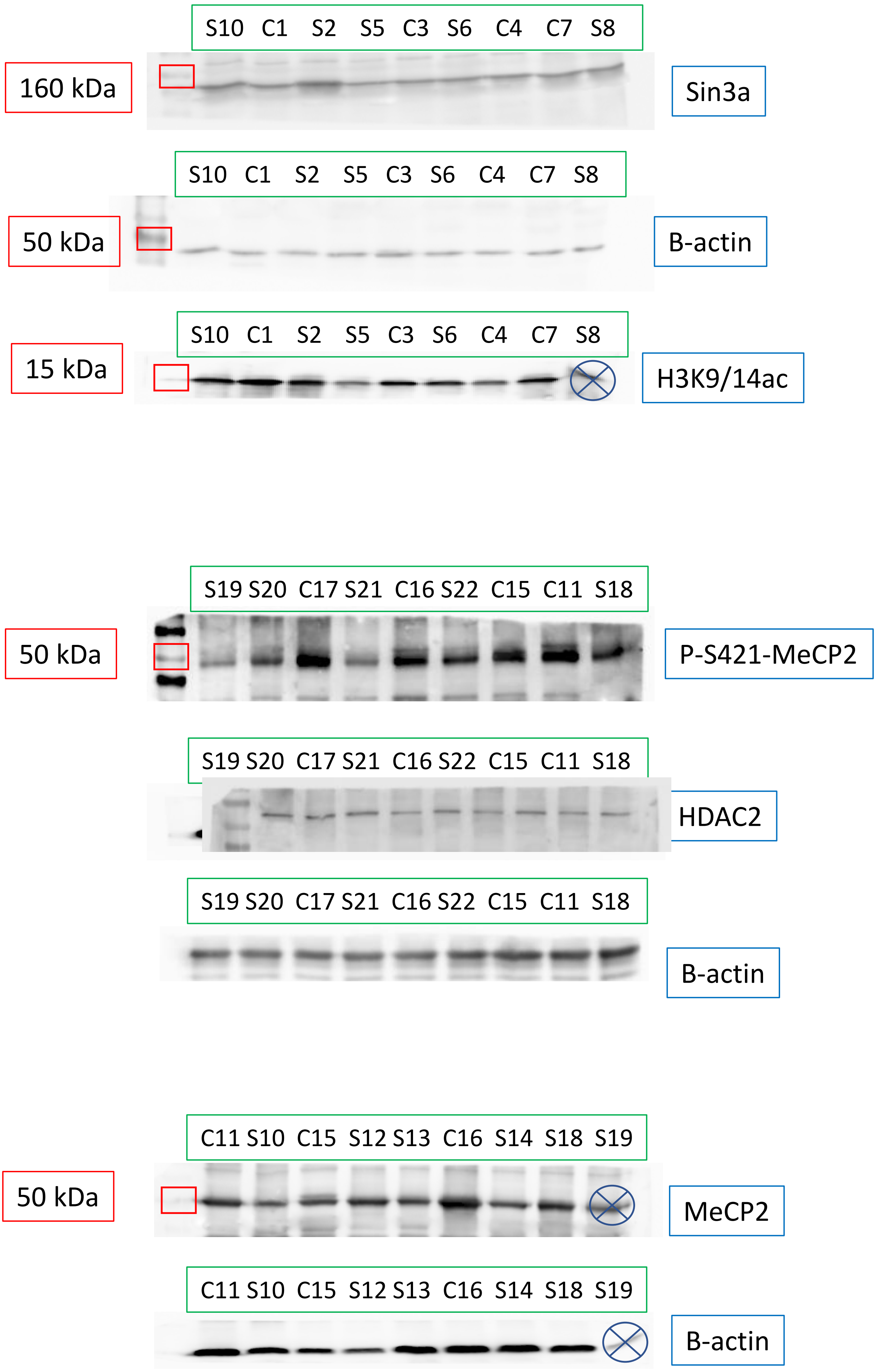

HIPPOCAMPUS

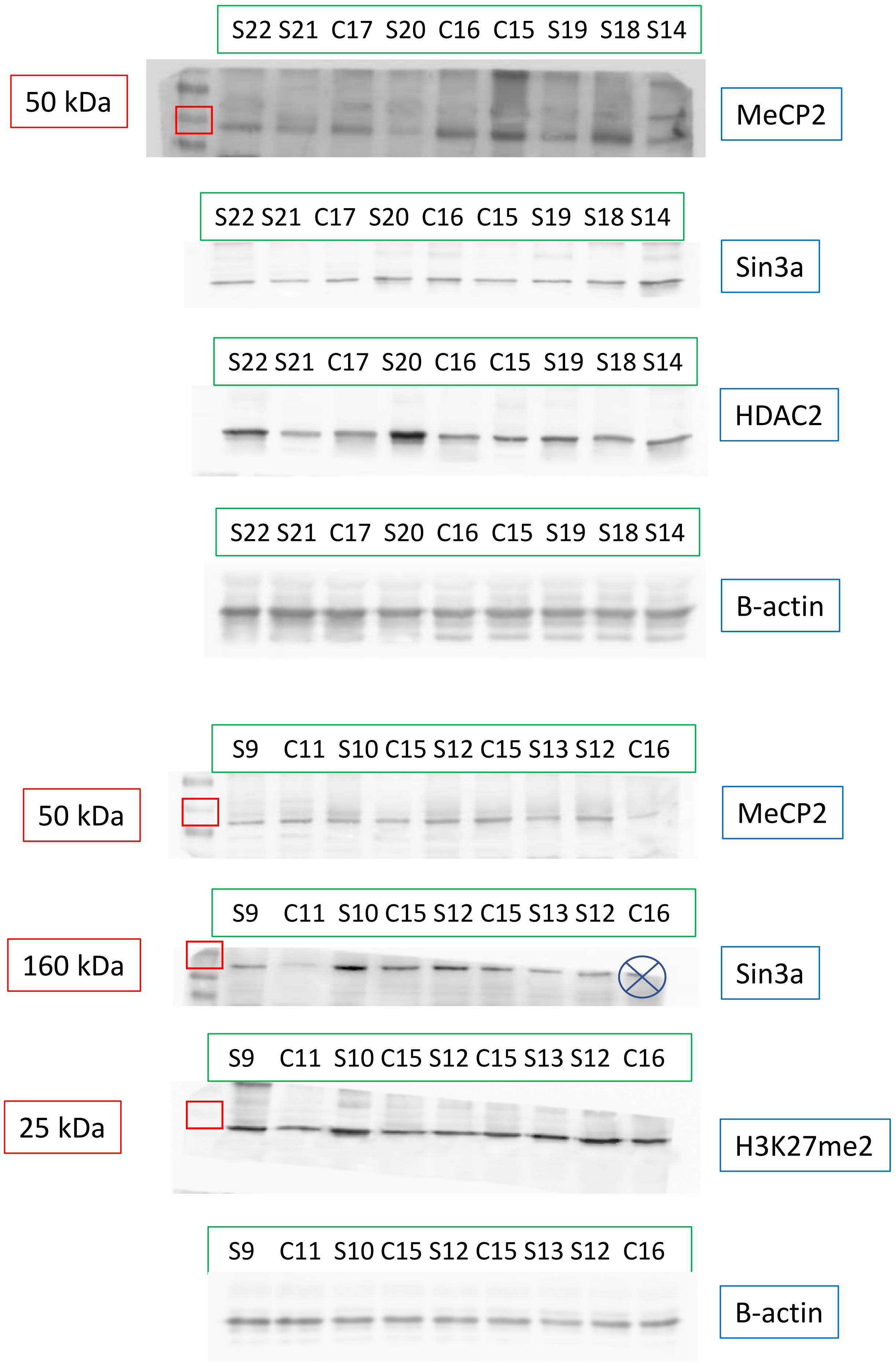

HIPPOCAMPUS

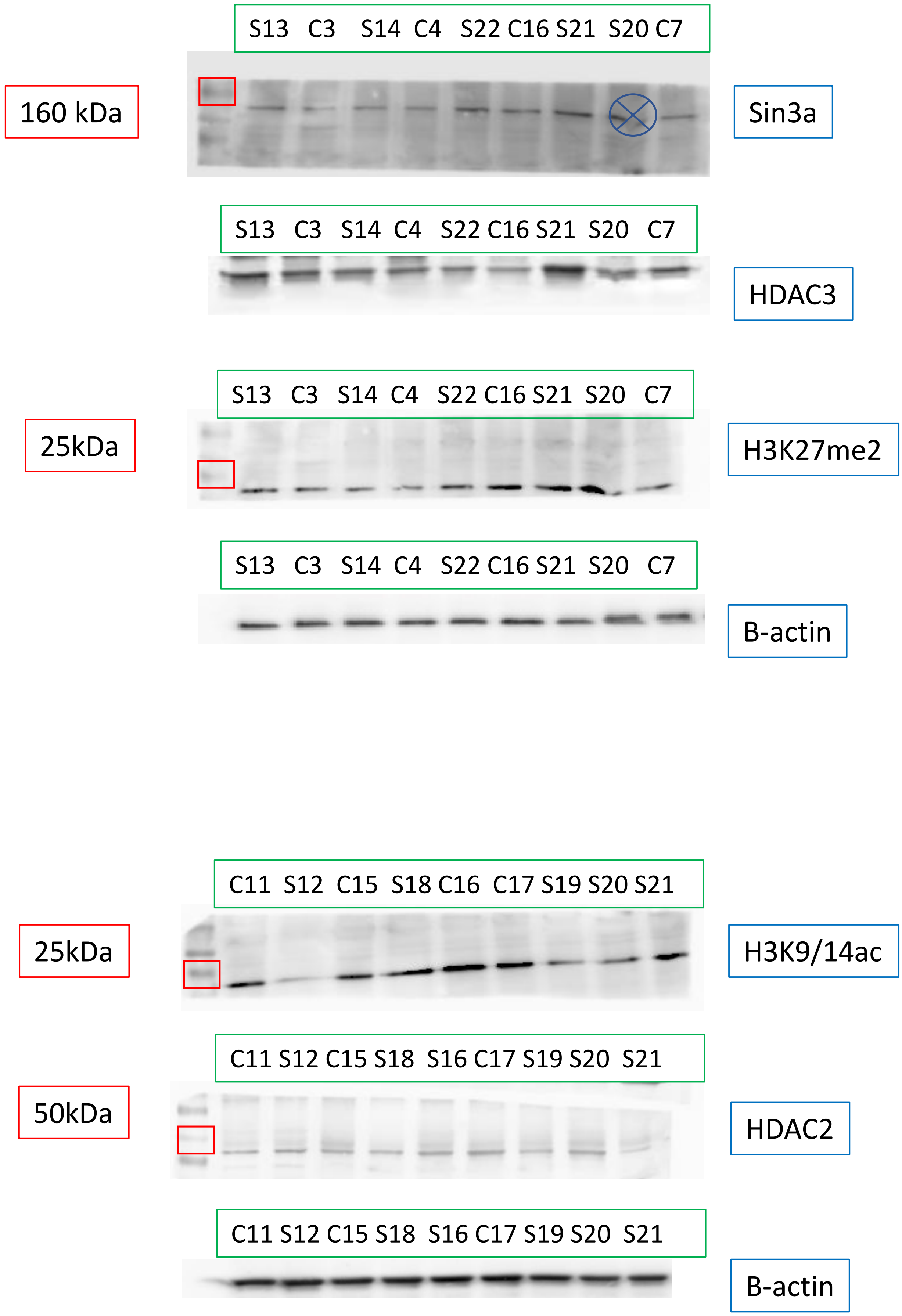

HIPPOCAMPUS

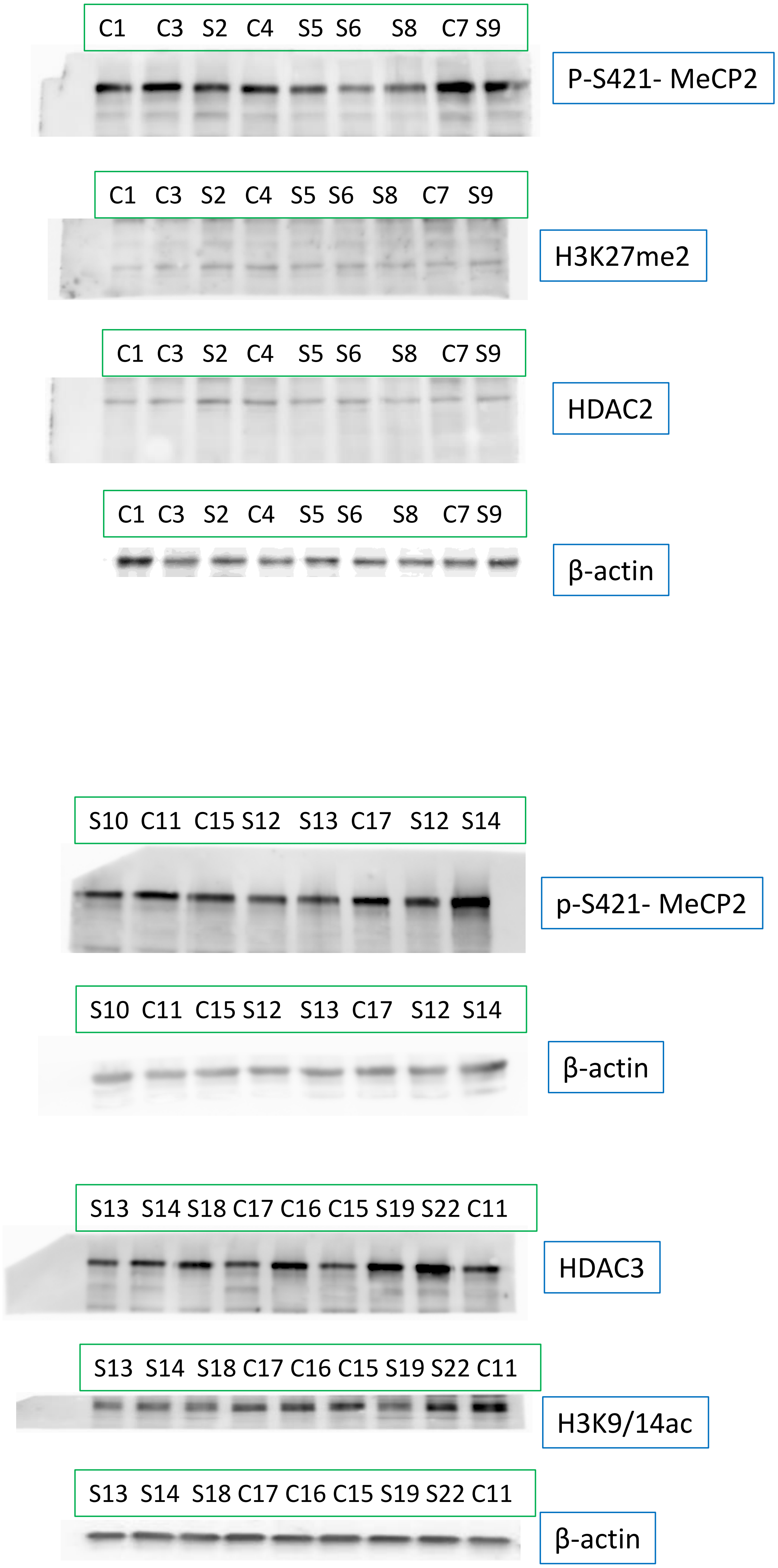

HIPPOCAMPUS

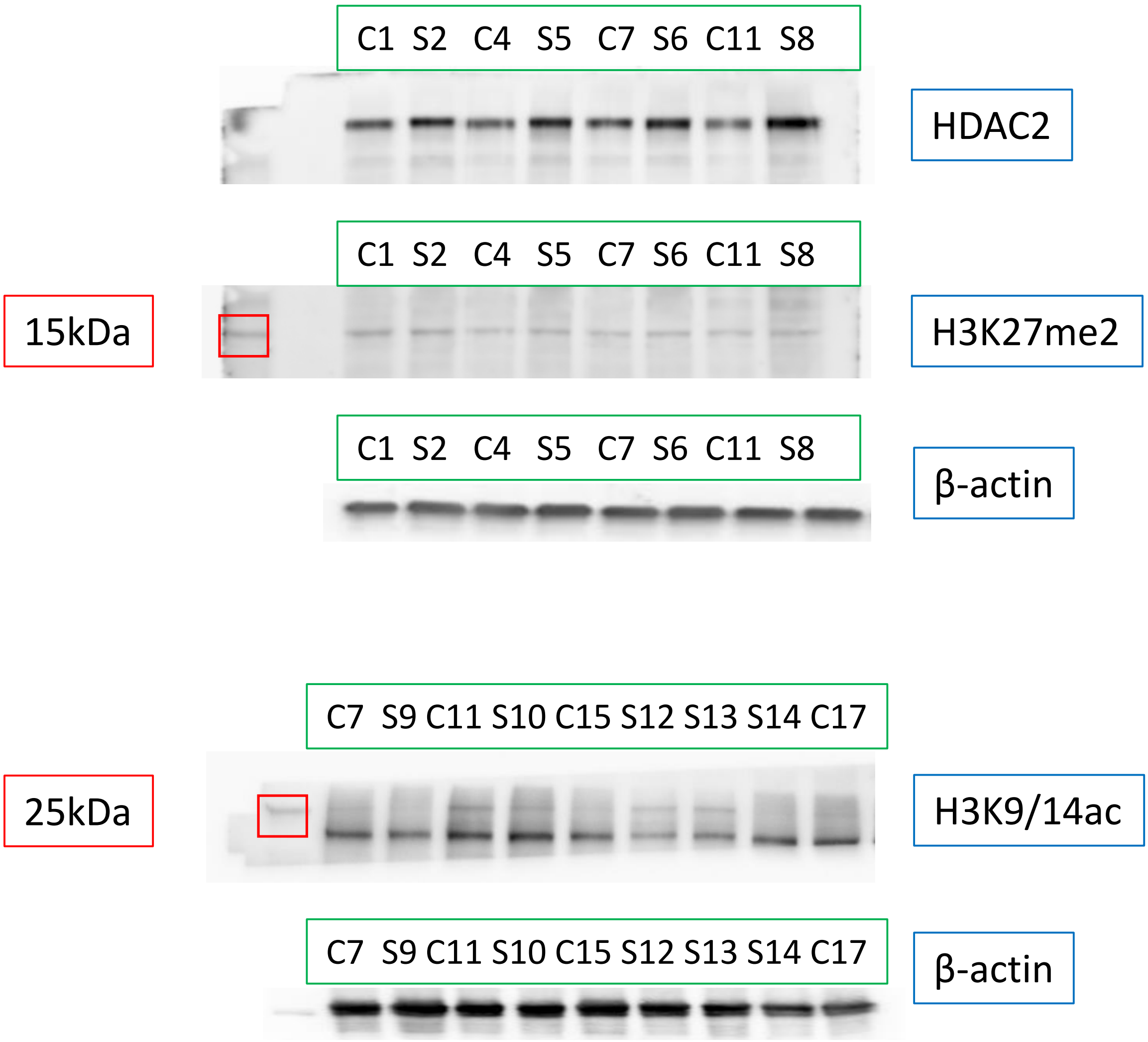

FRONTAL CORTEX

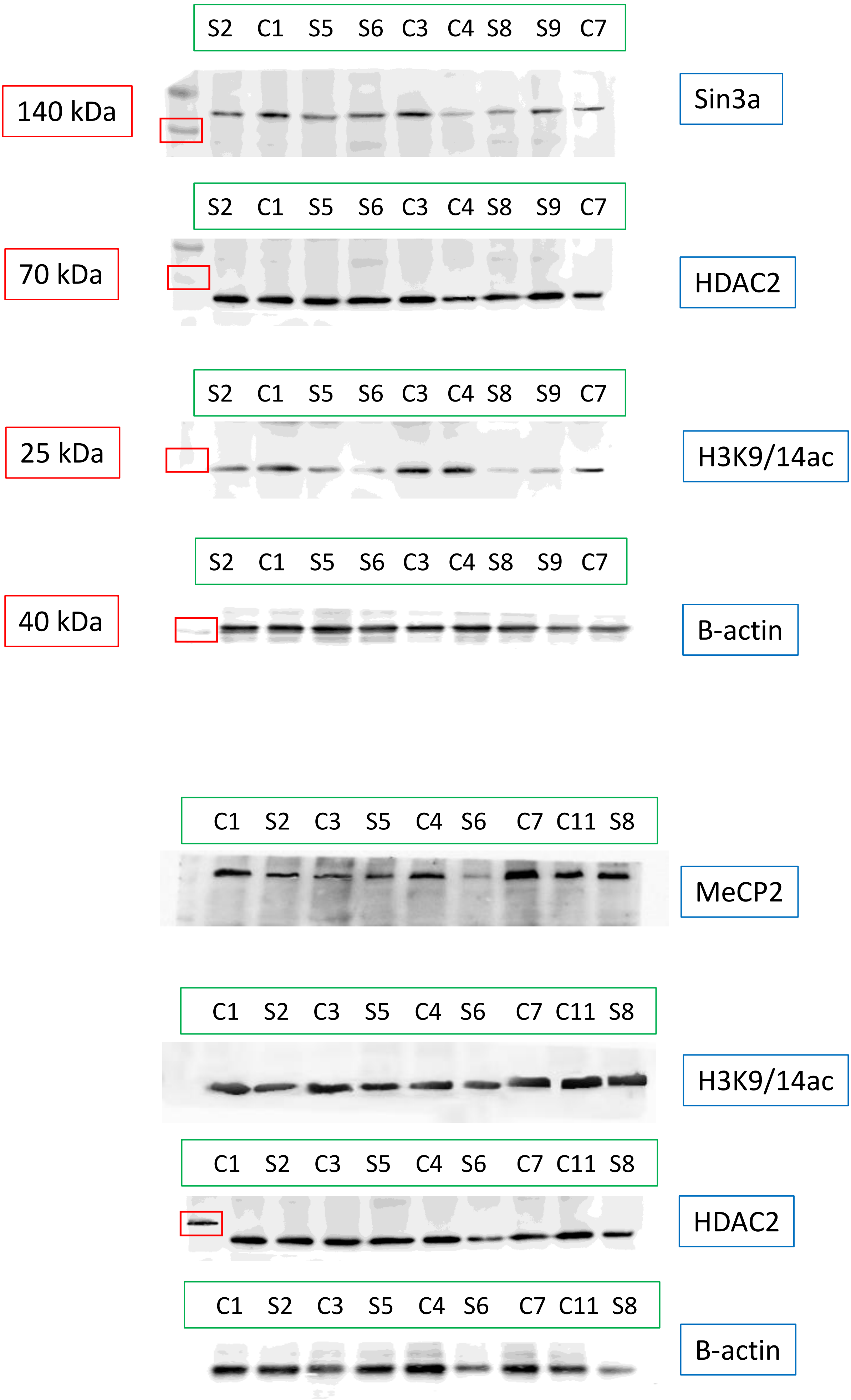

FRONTAL CORTEX

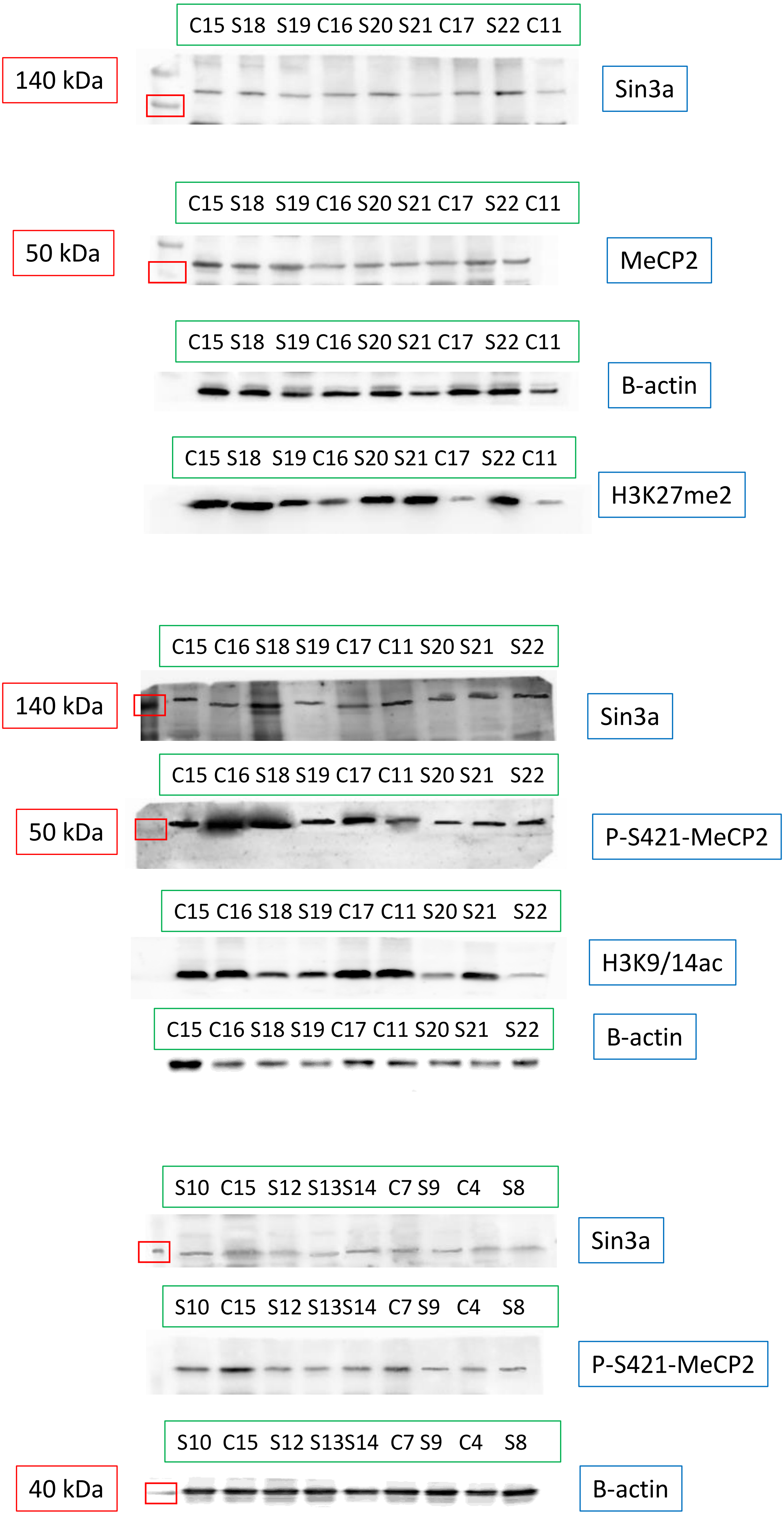

FRONTAL CORTEX

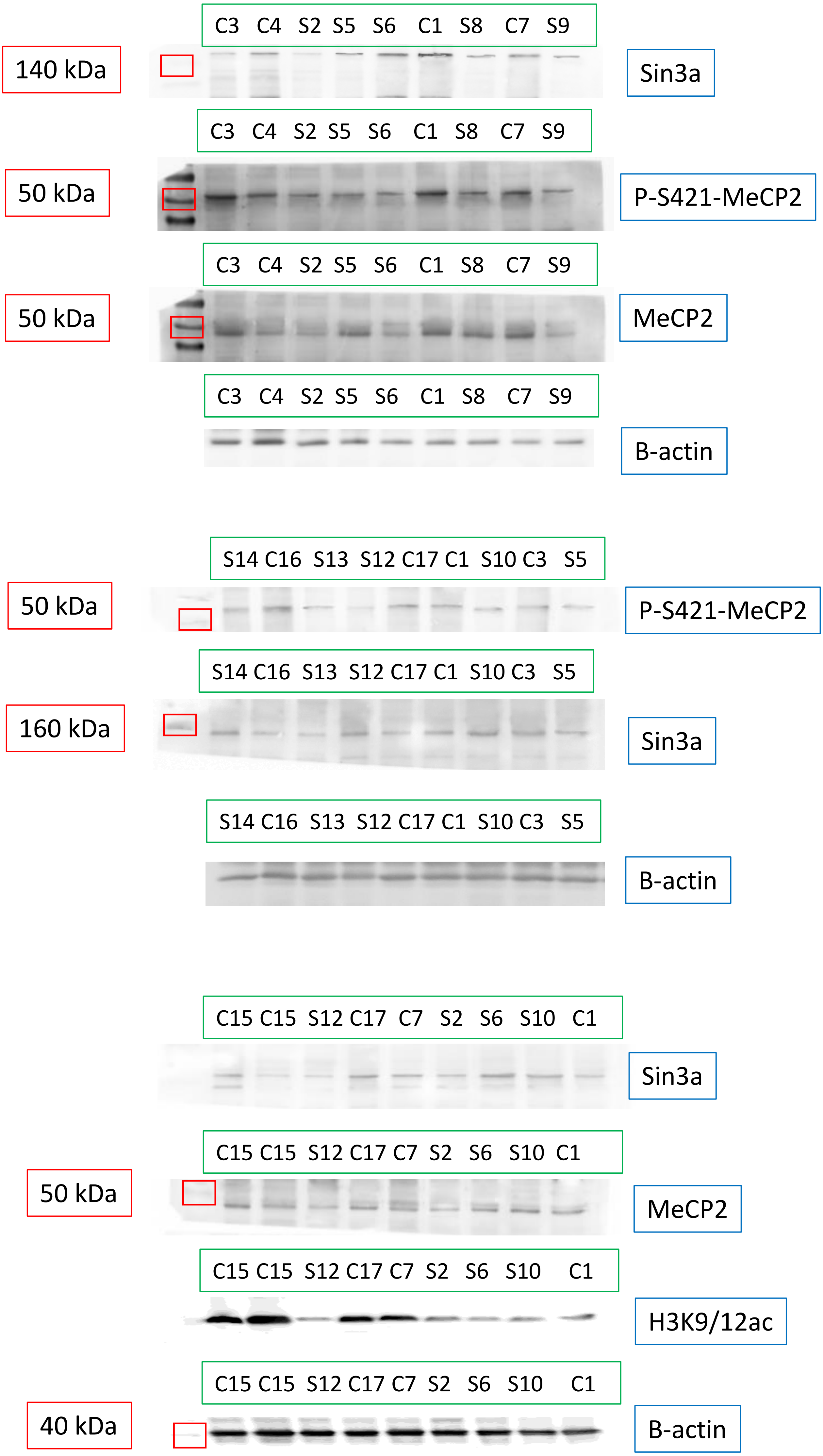

FRONTAL CORTEX

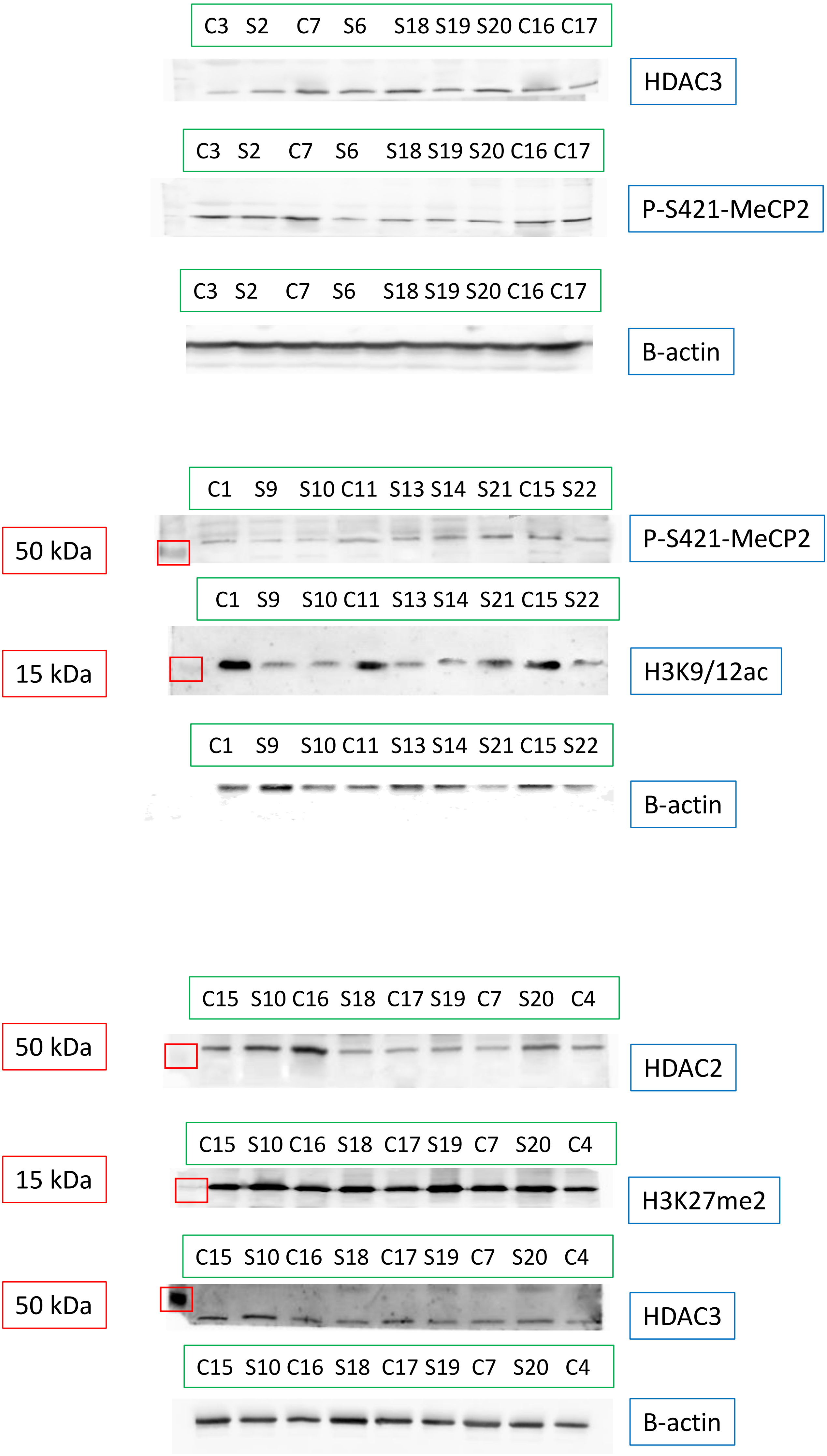

FRONTAL CORTEX

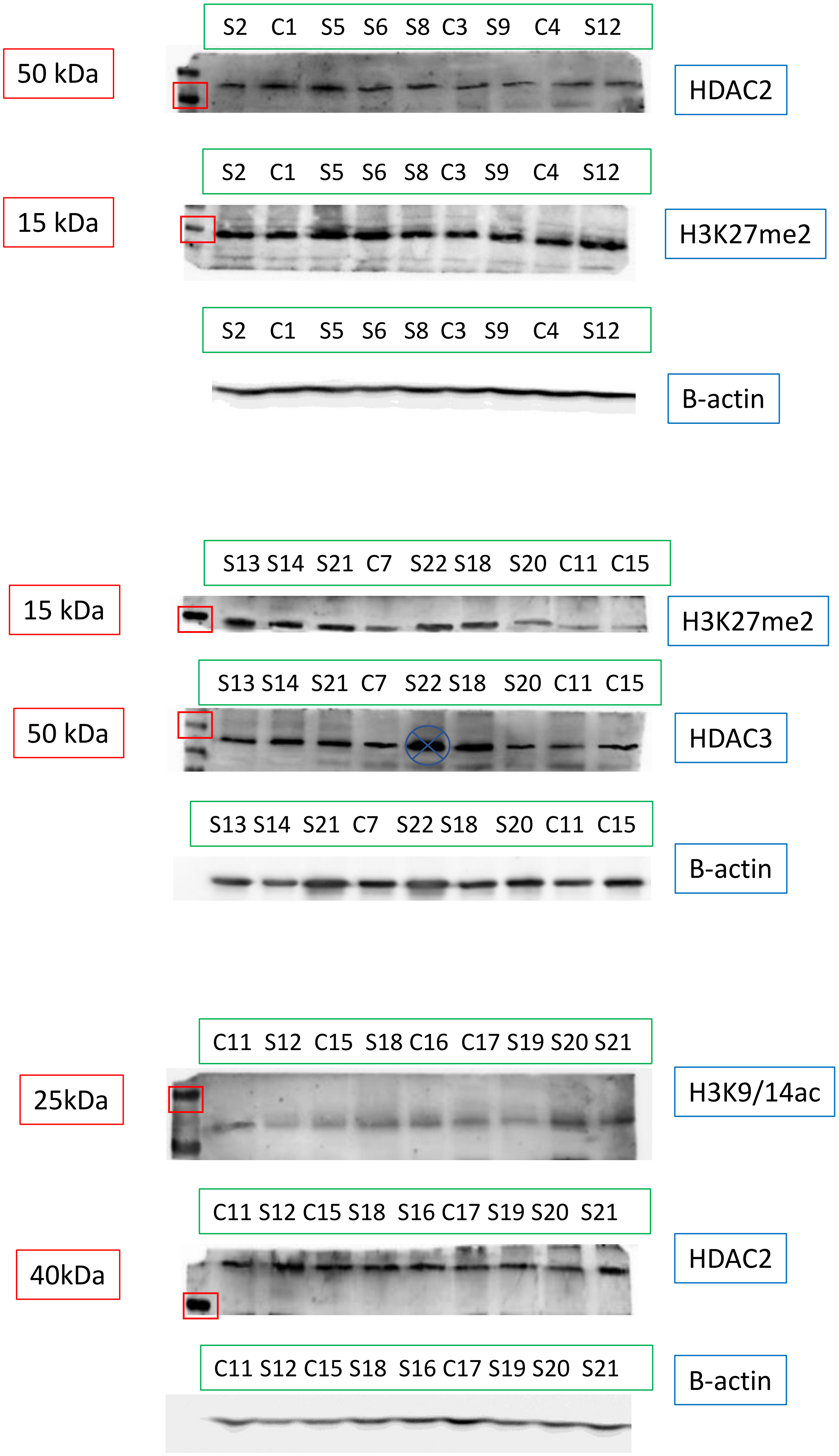

FRONTAL CORTEX

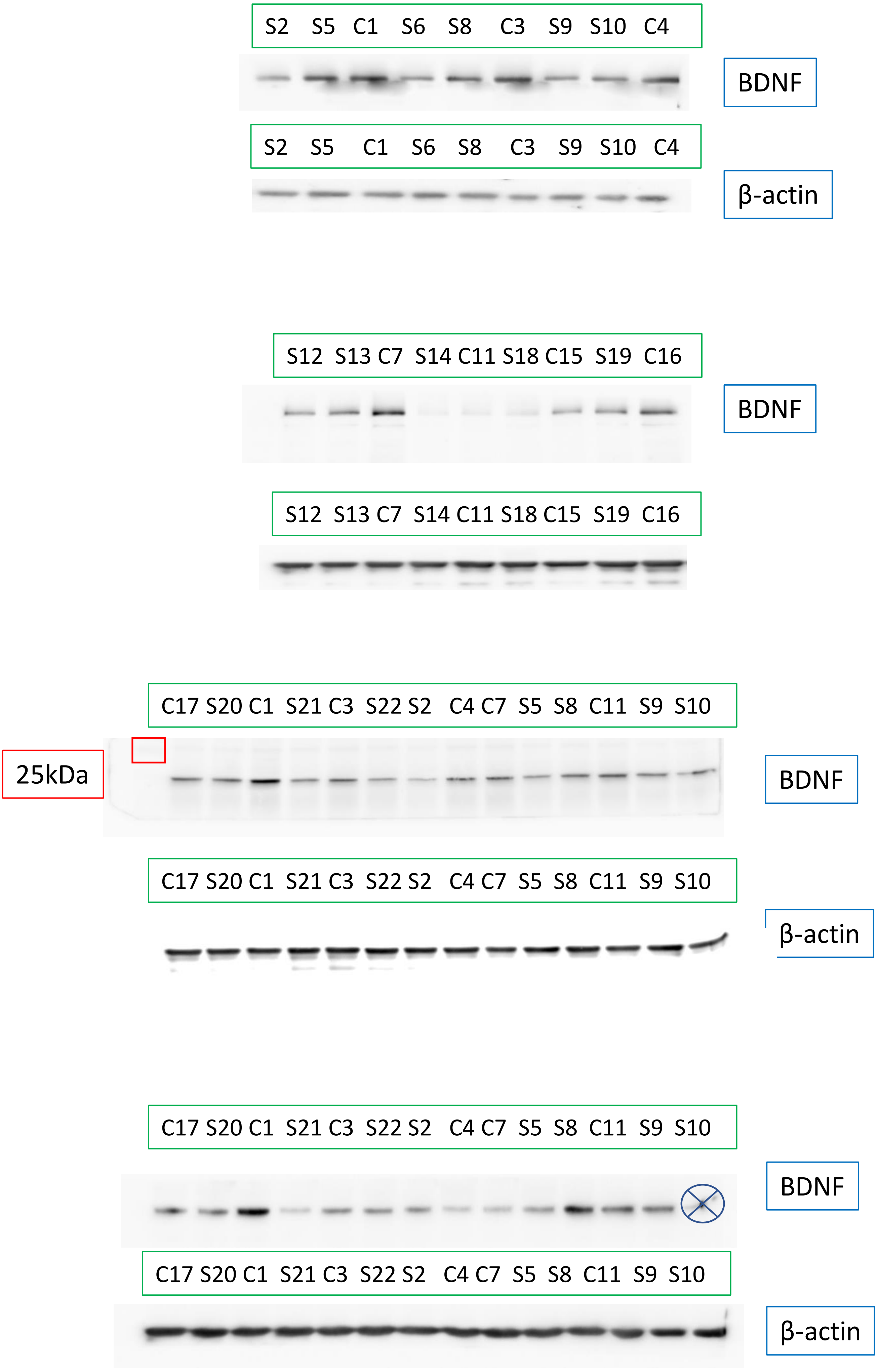

FRONTAL CORTEX

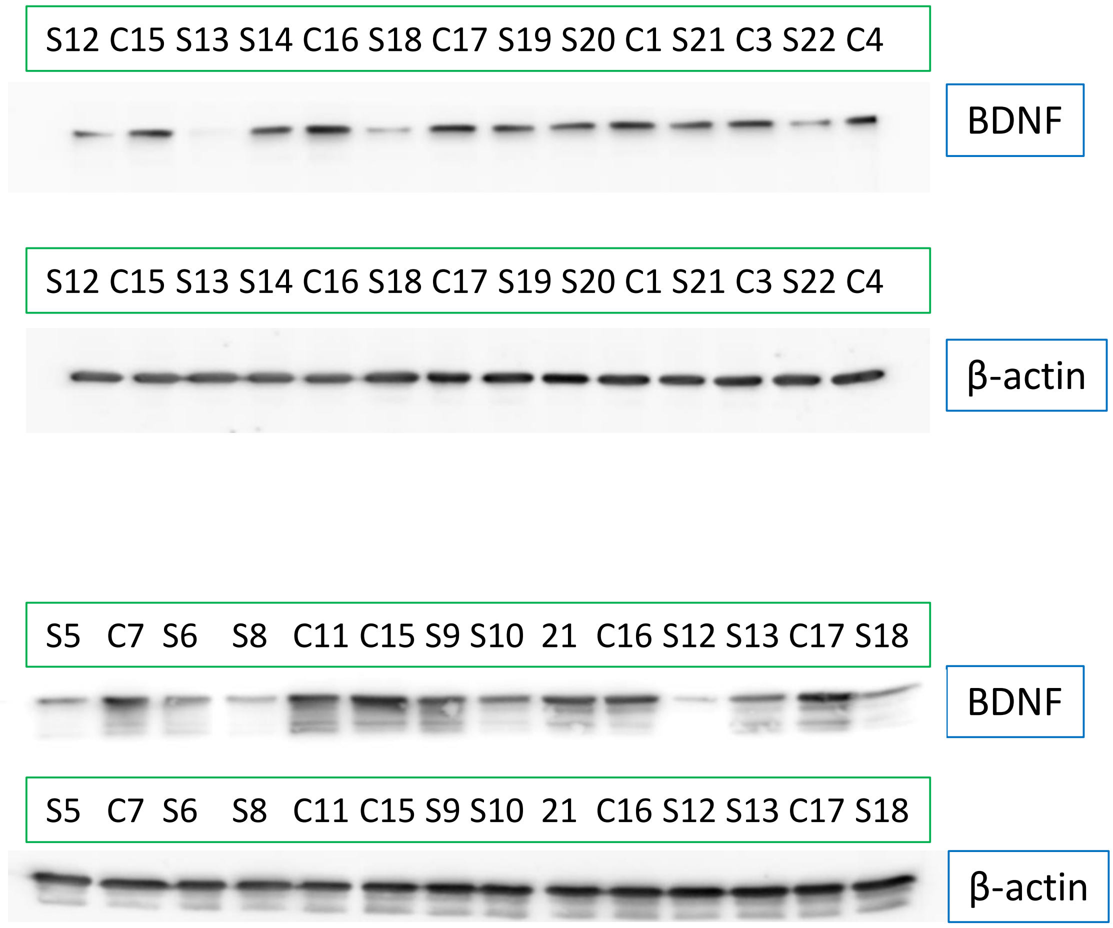

Supplement: S1 Raw images — (PDF) [file pone.0239335.s001.pdf]
